# Supplementary material for: The Structure and Chemical Composition of the Cr and Fe Pyrolytic Coatings on the MWCNTs’ Surface According to NEXAFS and XPS Spectroscopy
Source: Nanomaterials (Basel). 2020 Feb 21;10(2):374. doi: 10.3390/nano10020374 (PMC7075293; doi:10.3390/nano10020374)
Supplement: Supplementary file 1 [file nanomaterials-10-00374-s001.pdf]

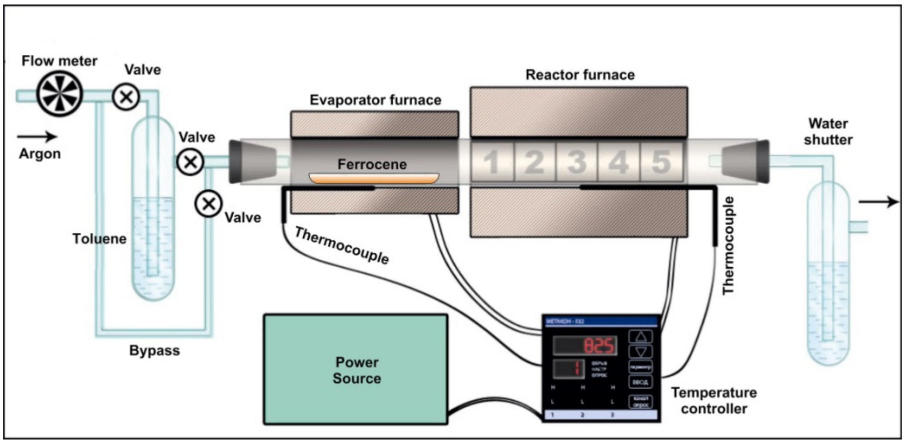

Figure S1. MOCVD-reactor for initial MWCNTs synthesis.

The installation for MWCNTs synthes consists of a cylindrical quartz reactor (length - 600 mm, outer diameter - 24 mm, inner diameter - 20 mm), placed in a system of two successive furnaces (Figure S1). The evaporation furnace and the reaction furnace with thermocouples located inside are connected to the power source through the METACON-532 temperature controller. At high temperatures (800–850 °C), the pyrolysis furnace has a uniform (± 2 °C) temperature distribution only in the middle reaction zone (region 3 in Figure S1), the length of which is 50 mm, and the length of the furnace is 250 mm. Inside the cylindrical reactor, in the zone of the pyrolysis furnace, 5 cylindrical quartz liners 50 mm long, with an external diameter of 19 mm and an internal diameter of 17 mm are placed. At the entrance to the main chamber of the reactor, a system for bubbling and supplying an inert gas was implemented. To exclude the possibility of atmospheric air entering the reactor volume an outlet water shutter was used. The argon flow, controlled by a high-precision gas flow meter AALBORG GFS17, entered the bubbler with toluene and transferred toluene vapor to the zone of the evaporation furnace. Ferrocene vapors were sublimated from a quartz boat placed in this zone with ferrocene and mixed with toluene vapors in an argon stream. The vapor mixture was transferred further to the zone of the pyrolysis furnace. In the central zone of the pyrolysis furnace, the mixture of ferrocene and toluene vapors decomposed with the formation of MWCNT arrays on cylindrical quartz inserts. The temperature of the ferrocene evaporator furnace, the temperature of the pyrolysis furnace of a mixture of ferrocene and toluene vapor, and the argon flow rate were selected so that the MWCNT arrays obtained in the third zone of the pyrolysis furnace had equal wall thickness along the entire length of the deposited nanotube array. In order to obtain cylindrical arrays of MWCNTs with equal walls thickness the pyrolysis temperature was 825 °C and the synthesis time - 4 hours. After the synthesis, the furnaces were cooled to room temperature, the reactor was opened and the quartz insert with the MWCNT array deposited inside was removed from the third pyrolysis zone. Then, the MWNT array was mechanically separated from the quartz liner and milled to a powdery homogeneous state suitable for subsequent studies and synthesis of hybrid materials based on MWNTs.

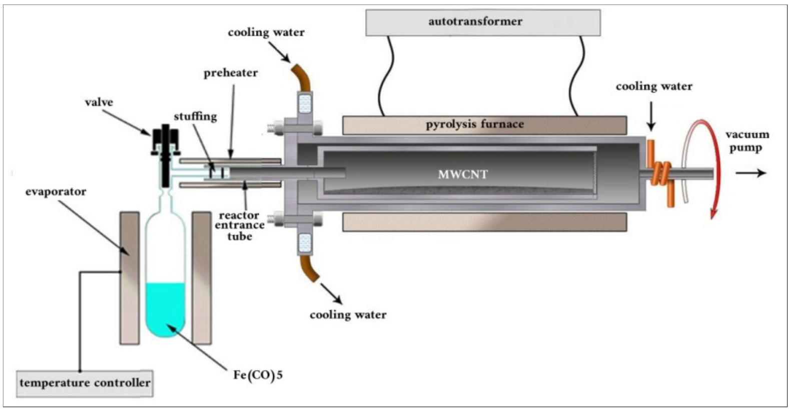

Figure S2. MOCVD-reactor for Fe/MWCNTs composite synthesis.

The scheme of the installation for the deposition of pyrolytic iron on the surface of the MWCNTs consists of the reaction volume with a front flange which is fastened to the volume with the six stud bolts (Figure S2). Stainless steel tube welded to the center of the flange. On the one hand, the tube through rubber seals is connected to the MOC evaporator, and on the other hand it enters the reaction volume. A 1 g portion of MWCNT was placed in a removable cylindrical liner, and the side hole was closed with a metal mesh to prevent loss of MWCNT. Then, the cylindrical liner was mechanically attached to the front metal flange body, connected by means of a cylindrical tube to the MOC evaporator.

Since iron pentacarbonyl is a liquid, an ampoule with a Teflon valve without an additional heating element was used as an evaporator. A certain volume of $Fe(CO)_5$ (1-24 g) in an argon stream was poured into an ampoule, and the MOC evaporator was connected to the reactor through rubber gaskets. Then the inlet of the MOC evaporator was opened, and the reactor with the evaporator was pumped out using a fore-vacuum pump through a liquid nitrogen cold trap to a residual pressure of 1.33 Pa. Next, the reactor was rotated at a speed of 60 rpm for about 1 hour while continuously pumping the reactor using a vacuum pump and heating the reactor to a temperature of 2400C. When the set temperature was reached, the teflon valve on the ampoule was opened and $Fe(CO)_5$ vapors were fed into the reactor. The evaporation rate of iron pentacarbonyl was controlled by the degree of valve opening on the ampoule; evaporation was carried out at room temperature. After a certain time (0.5-1.5 hours), the valve of the $Fe(CO)_5$ evaporator was closed and the heating of the pyrolysis furnace was turned off. The reactor was cooled to room temperature, argon was injected, then the reactor was opened and the resulting hybrid nanomaterial (pyrolytic Fe)/MWCNTs was unloaded. Then, the MWNT array was mechanically separated from the quartz liner and milled to a powdery homogeneous state suitable for subsequent studies and synthesis of hybrid materials based on MWNTs.

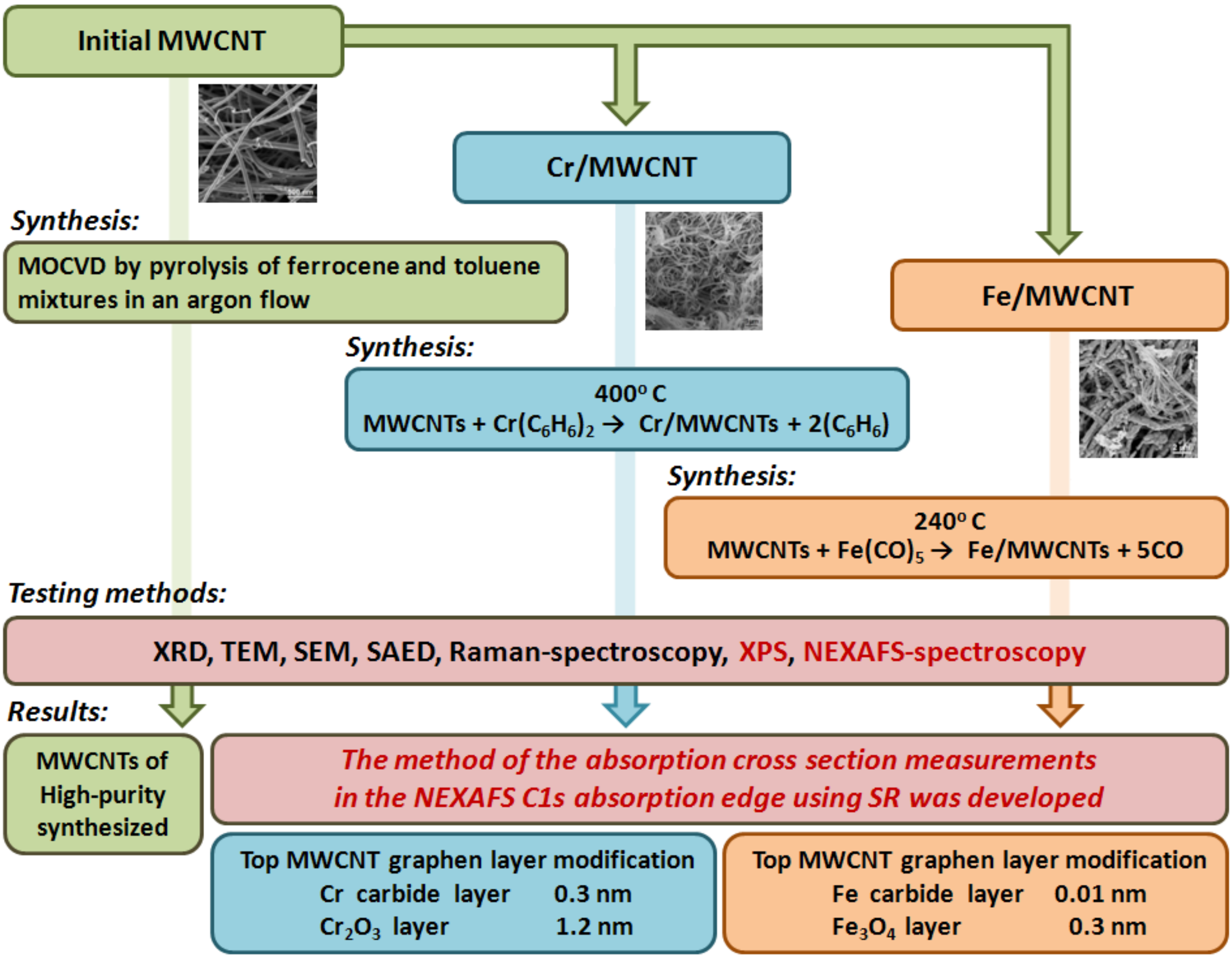

Figure S3. Scheme of all processing stages

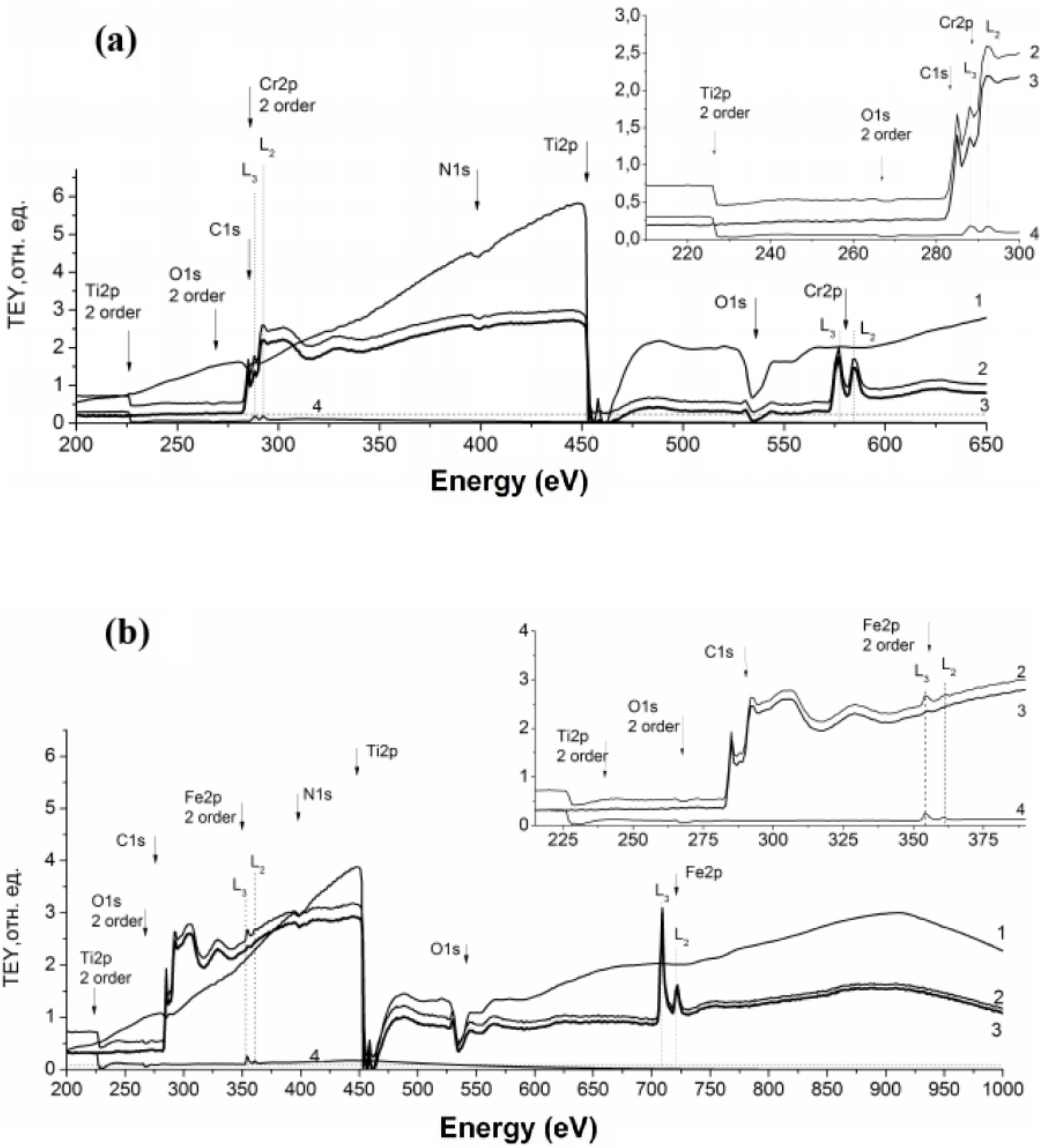

Figure S4. Spectral dependences of the TEY signal of Cr/MWCNT (a) and Fe/MWCNT (b) nanocomposites using a Ti filter without taking into account background radiation (2) and monochromatic signal (3), taking into account long-wave (dashed line) and second-order (4) background radiation, I0E0φ(E0) spectral dependence (1) in a wide and narrow (in the inset) energy range. The arrows indicate the atoms absorption edges positions, and the vertical dashed lines indicate the structure in the Cr2p and Fe2p regions of the absorption edges in the first and second diffraction orders.

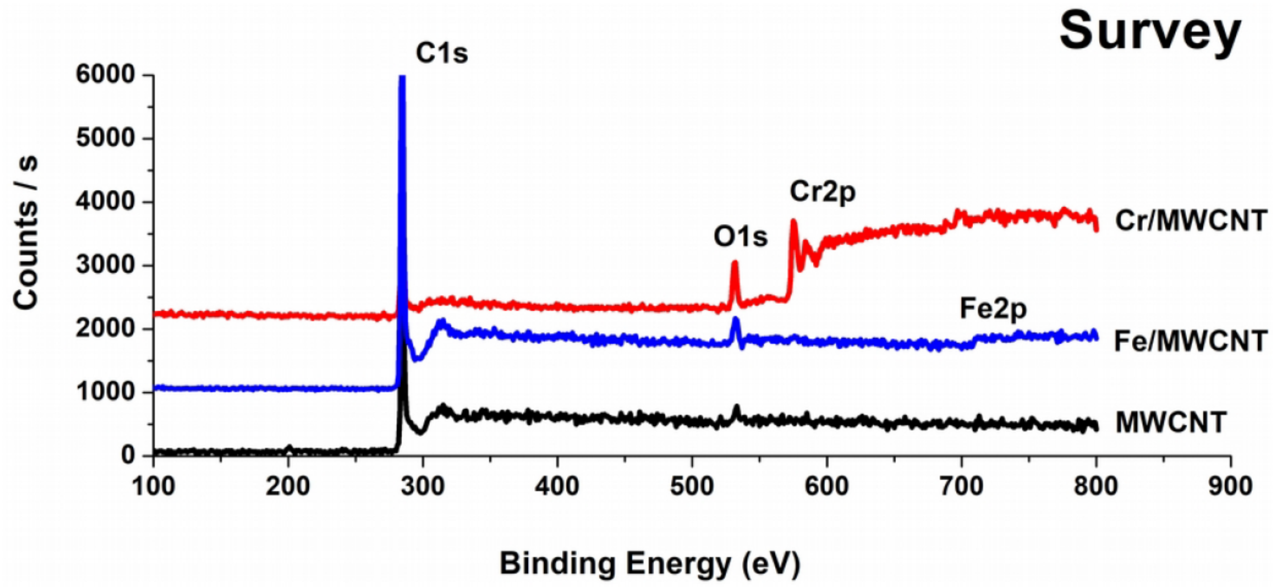

Figure S5. XPS spectra of the nanocomposites and the initial MWCNTs in a wide spectral range.

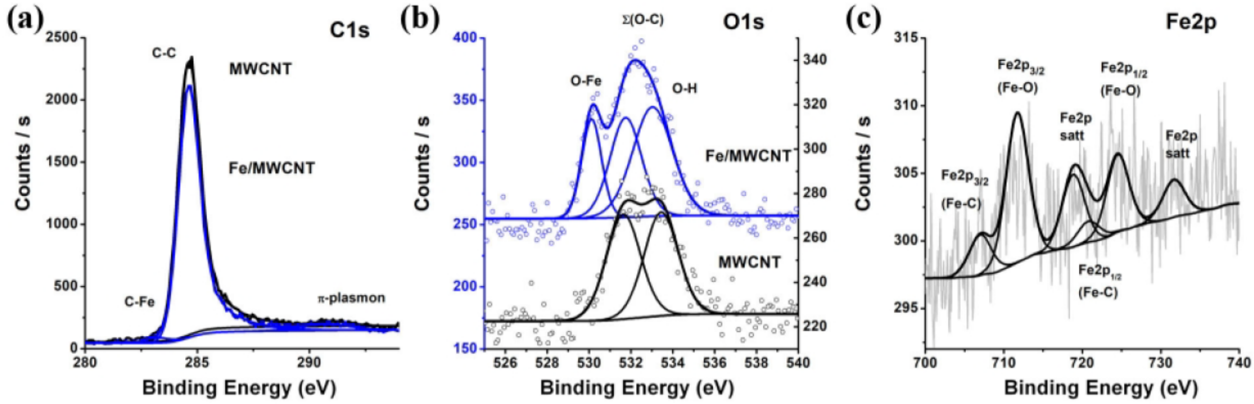

Figure S6. XPS spectra of the Fe/MWCNTs nanocomposite and initial MWC-NTs.
